# Severe Diabetic Ketoacidosis With Fatal Cerebral Edema in an Adult

**DOI:** 10.7759/cureus.107254

**Published:** 2026-04-17

**Authors:** Sherley M Rosa-Santiago, Daniel E Font-Rivera, Ricardo Fernandez-Gonzalez, Samille Olivera-Latorre, Noel Torres-Santiago

**Affiliations:** 1 Pulmonary and Critical Care Medicine, San Juan City Hospital, San Juan, PRI; 2 Pulmonary and Critical Care Medicine, Hospital Menonita Caguas, Caguas, PRI; 3 Nephrology, USF Health, Tampa, USA; 4 Diabetes and Endocrinology, University of Puerto Rico, Medical Sciences Campus, San Juan, PRI

**Keywords:** acute fulminant cerebral edema, acute respiratory failure, brain herniation, diabetes, diabetic ketoacidosis, type 1 diabetes mellitus

## Abstract

Diabetic ketoacidosis (DKA) is a life-threatening complication of type 1 diabetes, and cerebral edema is well described in children; it is rare in adults and carries a high mortality. We present the case of a 47-year-old male with poorly controlled type 1 diabetes who was brought to the ED unresponsive. On arrival, he was obtunded, required intubation, and vasopressor support. Laboratory evaluation was consistent with severe DKA, including marked hyperglycemia, high anion gap metabolic acidosis, and ketonemia. He also presented with multiple electrolyte disturbances, including hyperkalemia, hyperphosphatemia, and acute kidney injury. He was started on standard DKA management; however, imaging revealed diffuse cerebral edema with signs concerning for herniation. Despite initiation of hypertonic therapy, his neurological status worsened, and repeat imaging showed progression of cerebral edema with findings suggestive of pseudo-subarachnoid hemorrhage. The patient died within 72 hours of admission. This case highlights the severe course that DKA can take when complicated by cerebral edema and emphasizes the importance of early recognition and close monitoring.

## Introduction

Diabetic ketoacidosis (DKA) is one of the most serious acute complications of type 1 diabetes mellitus and remains a significant cause of morbidity in patients with poor glycemic control [1,2]. Although cerebral edema is a well-recognized complication of DKA in children, it is rare in adults [3-5]. When it occurs in adults, it is associated with significantly higher mortality compared to pediatric cases [3,4]. The pathophysiology of cerebral edema in DKA is not fully understood and is likely multifactorial, with proposed mechanisms including osmotic disequilibrium, cerebral hypoperfusion, and rapid shifts in plasma osmolality during treatment [5,6]. In addition, treatment-related factors such as aggressive fluid resuscitation and rapid correction of hyperglycemia may worsen intracellular fluid shifts, leading to cerebral swelling [3].

In this case report, we present a 47-year-old male who arrived in severe DKA with multiorgan failure and was found to have diffuse cerebral edema on neuroimaging upon admission. Despite aggressive intensive care management, the patient suffered progressive neurological deterioration and died within 72 hours. Considering the rarity and high mortality of cerebral edema in adults with severe DKA, this case is presented to highlight its clinical course, imaging progression, and key considerations for early recognition and management.

## Case presentation

A 47-year-old man with a history of Hodgkin lymphoma, diagnosed at age 16 and in remission for approximately 20 years, and type 1 diabetes mellitus secondary to prior chemotherapy, presented in August 2023 to the ED after being found unresponsive at home. His ex-girlfriend reported that she had been trying to contact him for a day without success and therefore performed a wellness check. Upon arriving at his residence, she observed him through a window, leaning forward against the refrigerator, unresponsive. She contacted the patient’s father, and both entered the home with help from a neighbor. They found the patient with eyes open but obtunded, not reacting to verbal or painful stimulation. His ex-girlfriend explained that he had been vomiting for the past two days, mostly gastric contents, and she had been bringing him electrolyte drinks without improvement. She believed the vomiting started after eating “bad food.”

The father described the patient as having very poor adherence to diabetes management since diagnosis. He rarely monitored his blood glucose and relied on symptoms such as dizziness to assume hypoglycemia or hyperglycemia. According to the father, the patient had suffered multiple episodes of syncope caused by hypoglycemia that were treated at home with sugar packets. He also reported frequent episodes of hyperglycemia above 300 mg/dL that were occasionally corrected with insulin but without visiting a doctor. The father also noted progressive bilateral visual loss and generalized weakness developing over the past three months. The family denied prior episodes of DKA or recent medical evaluation. They also denied that he had recently complained of fever, chills, abdominal pain, chest discomfort, trauma, dyspnea, dysuria, or toxic ingestions.

Upon arrival at the ED, the patient was hypotensive with a blood pressure of 90/36 mmHg, tachycardic with a heart rate of 91 bpm, and normothermic. He had a Glasgow Coma Scale score of 3 and was immediately intubated for airway protection. The initial physical examination showed an acutely ill male, intubated, with pinpoint pupils and bilateral periorbital edema. The oral mucosa was extremely dry. The lungs had symmetric expansion and were clear to auscultation. The abdomen was soft and nontender with normal bowel sounds. There were no skin lesions, ulcers, or peripheral edema. Neurological responses were absent, including no response to painful stimuli.

Plasma glucose was above the measurable range (>1000 mg/dL), serum bicarbonate was severely reduced at 9.8 mmol/L, and arterial pH was 6.882. The arterial blood gas demonstrated a high anion gap metabolic acidosis with superimposed severe respiratory acidosis. Using admission electrolytes (Na 131 mEq/L, Cl 95 mEq/L, HCO₃ 7 mEq/L), the anion gap was calculated at 29 mEq/L, with a delta ratio of 1.0, consistent with a high anion gap metabolic acidosis without additional metabolic disturbances. The measured serum osmolality was 294 mOsm/kg. However, when corrected for severe hyperglycemia, the sodium was approximately 149 mEq/L, and the effective serum osmolality was approximately 329 mOsm/kg, reflecting a significant hyperosmolar state. The patient also had severe hyperkalemia (8.7 mmol/L) and hyperphosphatemia (12 mg/dL). Renal function was markedly impaired, with a creatinine level of 5.46 mg/dL, a BUN of 80 mg/dL, and an estimated creatinine clearance of 21 mL/min. Troponin was elevated at 1808 ng/L and increased above 8000 ng/L on repeat evaluation, likely indicating demand ischemia. Creatine kinase was mildly elevated. Urinalysis demonstrated ketonuria, glucosuria, and granular casts. Serial arterial blood gas measurements demonstrated gradual improvement in metabolic parameters over time (Table 1).

**Table 1:** Serial arterial blood gas values during hospitalization Day 1 corresponds to hospital admission.

Parameter	On arrival	Day 1 (AM)	Day 1 (PM)	Day 2 (AM)	Day 3 (AM)	Day 4 (AM)	Day 5 (AM)	Reference range
pH	6.882	7.138	7.307	7.395	7.309	7.329	7.345	7.35-7.45
PaCO₂ (mmHg)	52.6	27.1	33.2	35.4	44.6	41.0	38.9	35-45
PaO₂ (mmHg)	205.4	156.7	107.8	65.3	72.1	60.2	72.5	80-100
HCO₃⁻ (mmol/L)	9.8	9.0	16.3	21.3	21.9	21.1	20.8	22-28

**Figure 1:**
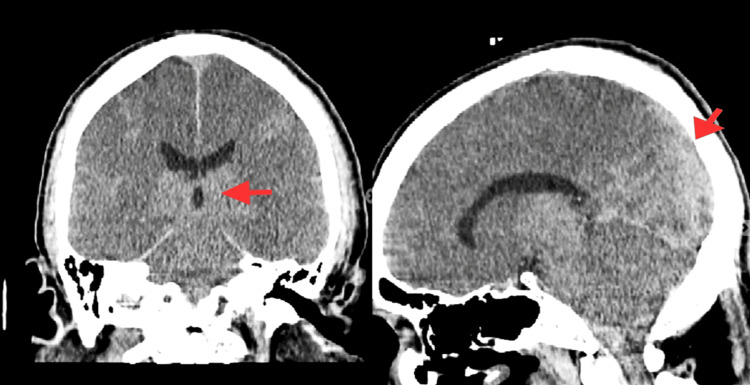
Initial non-contrast CT brain at admission showing diffuse loss of gray-white differentiation and slit-like ventricles, concerning for early cerebral edema Red arrows indicate areas of cerebral swelling.

A non-contrast head CT obtained on arrival showed diffuse loss of gray-white matter differentiation, a slit-like fourth ventricle, and caudal displacement of the cerebellar tonsils into the foramen magnum. These findings were strongly suggestive of diffuse cerebral edema with possible early herniation (Figure 1). The patient was started on multiple vasopressors, including norepinephrine, vasopressin, and epinephrine, due to persistent hypotension and a mean arterial pressure below 65 mmHg.

**Figure 2:**
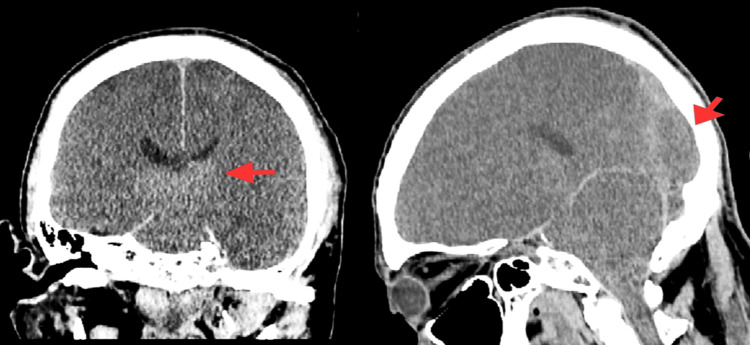
Follow-up non-contrast CT brain showing progression of diffuse cerebral edema, characterized by loss of gray-white differentiation, sulcal effacement, and ventricular compression Red arrows indicate areas of cerebral swelling.

Management of DKA with IV insulin therapy was initiated immediately. Management was consistent with current guideline-directed therapy for DKA [1]. The patient received an initial insulin bolus followed by continuous IV insulin infusion at 0.1 units/kg/hr. Due to the severity of DKA and the presence of renal dysfunction, early basal insulin was also administered. Aggressive fluid resuscitation was provided with four liters of 0.9% normal saline, followed by maintenance fluids. A sodium bicarbonate drip was initiated due to the severe metabolic acidosis and bicarbonate deficit estimated at 544 mEq. Calcium gluconate was administered for stabilization of the cardiac membrane due to hyperkalemia, and phosphate binders were started to address hyperphosphatemia. A repeat head CT performed 24 hours after admission showed worsening diffuse cerebral edema (Figure 2). Because of the profound neurological findings, hypertonic saline at 3% concentration was initiated for cerebral edema, and serum sodium levels were monitored closely every four hours. Serial laboratory values, including glucose, electrolytes, and renal function, are summarized in Table 2.

**Table 2:** Serial laboratory values, including metabolic panel and renal function Day 1 corresponds to hospital admission. BUN, blood urea nitrogen; Cr, creatinine; eGFR, estimated glomerular filtration rate

Parameter	On arrival	Day 1 (AM)	Day 1 (PM)	Day 2 (AM)	Day 2 (PM)	Day 3 (AM)	Day 3 (PM)	Day 4 (AM)	Day 5 (AM)	Reference range
Glucose (mg/dL)	1200	815	604	221	257	224	186	229	163	70-100
BUN (mg/dL)	80	83	72	54	47	45	46	55	60	7-20
Creatinine (mg/dL)	5.46	5.26	4.44	3.82	3.39	3.01	2.83	3.30	3.51	0.6-1.3
BUN/Cr ratio	14.6	15.78	16.22	14.14	13.86	14.95	16.25	16.67	17.09	10-20
Sodium (mEq/L)	131	136	145	151	148	149	148	151	153	135-145
Potassium (mEq/L)	8.7	3.0	3.8	4.2	4.2	3.7	3.8	4.1	3.9	3.5-5.0
Chloride (mEq/L)	95	102	111	122	118	120	123	122	125	98-106
CO₂/HCO₃⁻ (mEq/L)	7	13	19	21	22	22	20	24	23	22-28
Anion gap (mEq/L)	29	24.0	18.8	12.2	12.2	10.7	8.8	9.1	8.9	8-12
Calcium (mg/dL)	6.4	6.4	7.0	7.1	7.3	7.2	7.4	7.4	7.3	8.5-10.5
eGFR (mL/min/1.73 m²)	10	12	15	18	20	24	25	21	20	>60

**Figure 3:**
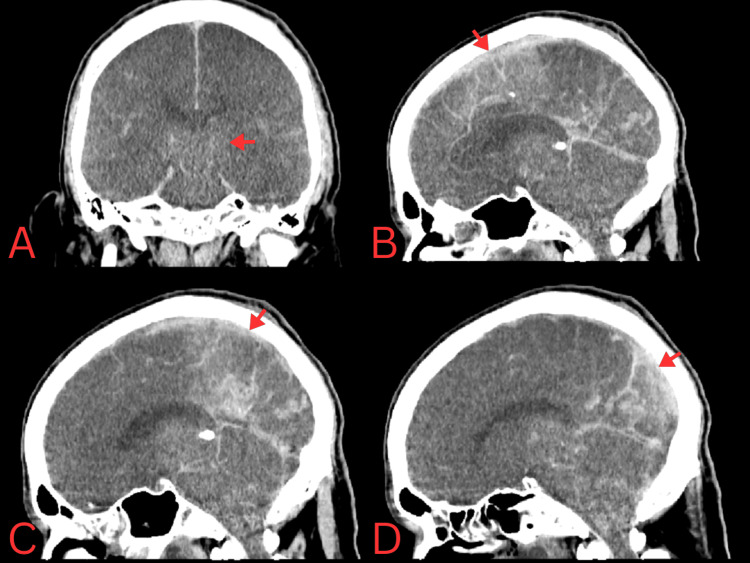
Third non-contrast CT brain demonstrating severe diffuse cerebral edema (A) Coronal view showing diffuse loss of gray-white matter differentiation and effacement of cerebrospinal fluid spaces, including the basal cisterns. (B-D) Sagittal views demonstrating diffuse cerebral swelling with sulcal effacement and compression of the ventricular structures. Hyperattenuation within the basal cisterns and cortical sulci was noted, raising concern for subarachnoid hemorrhage versus pseudo-subarachnoid hemorrhage in the setting of severe cerebral edema. Arrows indicate representative areas of cerebral swelling and hyperattenuation.

Neurosurgery evaluated the patient and determined that no surgical intervention was feasible due to the diffuse nature of the edema, evidence of early herniation, and absence of meaningful neurological responses. Over the following 48 hours, the patient remained critically ill despite intensive management. His metabolic parameters gradually improved, and the anion gap closed. However, neurological status did not recover. A repeat head CT performed 48 hours after admission showed a worsening appearance of symmetric hyperattenuation at the basal cisterns, concerning for pseudo-subarachnoid hemorrhage (Figure 3), a radiological sign commonly associated with severe cerebral swelling. After 72 hours of hospitalization, the patient experienced refractory shock and cardiac arrest and was pronounced dead.

## Discussion

Although DKA is a complication of type 1 diabetes, cerebral edema in adults is rarely encountered [3-5]. The exact pathogenesis of cerebral edema in DKA is not fully understood, and several mechanisms have been proposed [5,6]. When presentation occurs, mortality is extremely high, with reported rates ranging from approximately 20% to 40% in adult cases [7]. One mechanism is osmotic disequilibrium [5,6]. In chronic hyperosmolar states, the brain adapts by accumulating intracellular osmoles to protect against dehydration. When serum glucose declines rapidly during treatment, osmotic gradients shift, causing water to move intracellularly into brain tissue, leading to cytotoxic swelling [5,6].

Another proposed mechanism is cerebral hypoperfusion in the setting of severe dehydration [5]. Prolonged vomiting, poor oral intake, and shock can significantly reduce cerebral blood flow, as likely occurred in this patient. Subsequent restoration of perfusion during resuscitation may lead to reperfusion injury and endothelial dysfunction, contributing to vasogenic edema [5]. Additionally, severe metabolic acidosis, hyperketonemia, and inflammatory cytokines may increase blood-brain barrier permeability, allowing fluid to accumulate in the brain parenchyma [5,6]. Some adult cases of cerebral edema during DKA may also involve unrecognized hypoxic-ischemic injury prior to hospital presentation [3,4].

Previous reports of cerebral edema in adults with DKA describe severe metabolic derangements, delayed presentation, and poor neurological outcomes [3-5]. Consistent with these observations, our patient presented with prolonged symptoms, significant dehydration, and profound acidosis, all recognized risk factors for cerebral edema. However, unlike cases where neurological recovery has been reported with early recognition and intervention [8], this patient showed no improvement despite aggressive management, likely reflecting the extent of hypoxic and metabolic injury prior to presentation. In addition, prior reports have demonstrated that cerebral edema may develop or progress despite adherence to guideline-based therapy [7] and can present with imaging findings that mimic subarachnoid hemorrhage, further complicating diagnosis [9]. Severe neurologic complications may also extend beyond isolated cerebral edema, including syndromes such as posterior reversible encephalopathy syndrome [10].

In this case, the patient’s presentation of several days of vomiting, prolonged dehydration, severe acidosis, hemodynamic instability, and markedly elevated glucose likely contributed to a multifactorial process involving osmotic, ischemic, and inflammatory injury. CT findings of diffuse loss of gray-white differentiation and slit-like ventricles are consistent with cerebral edema and global hypoxic injury. Additionally, the patient’s last known well was more than 24 hours prior to presentation and intubation, suggesting a prolonged period of physiologic stress that likely contributed to the severity of brain injury.

Management of cerebral edema in DKA remains challenging, particularly in adults, given the limited evidence guiding therapy [5]. Hypertonic saline is often preferred, especially in patients with renal impairment. However, reversibility depends on the extent of the initial injury. Despite aggressive correction of acidosis, hypoxia, hypoperfusion, osmolar abnormalities, and hyperglycemia, this patient showed no neurological improvement, suggesting that the injury was likely irreversible at presentation. These findings further support the multifactorial pathophysiology of cerebral edema in DKA and underscore the importance of early recognition and timely intervention.

## Conclusions

This case highlights a rare but catastrophic complication of DKA in adults. Prolonged symptoms, severe dehydration, metabolic derangements, and delayed presentation likely contributed to the development of cerebral edema and a poor outcome. Clinicians should maintain vigilance for neurological deterioration in high-risk patients, with prompt neuroimaging and careful correction of metabolic abnormalities. Despite appropriate management, outcomes remain poor once severe cerebral edema and brainstem involvement occur. Clinicians should be aware of the risk of life-threatening neurological complications in severe DKA and closely monitor for the development of cerebral edema.
